# Analgesics for Dental Implants: A Systematic Review

**DOI:** 10.3389/fphar.2020.634963

**Published:** 2021-01-27

**Authors:** Matteo Melini, Andrea Forni, Francesco Cavallin, Matteo Parotto, Gastone Zanette

**Affiliations:** ^1^ Scholar at Oral Surgery and Implantology – Department of Biomedical and Neuromotor Science (DIBINEM), University of Bologna, Bologna, Italy; ^2^ Private Practice in Lodi, Bologna, Italy; ^3^ Independent Statistician, Solagna, Italy; ^4^ Department of Anesthesia and Pain Management and Interdepartmental Division of Critical Care Medicine, University of Toronto, Toronto, ON, Canada; ^5^ Department of Neurosciences and Anesthesiology, Dentistry Section, Chair of Dental Anesthesia, University of Padua, Padova, Italy

**Keywords:** systematic review, dental implant, analgesics, pain, post-operative pain

## Abstract

Postsurgical pain is commonly associated with dental and oral surgery, and the use of analgesics has been investigated in the management of postoperative pain. This systematic review summarizes available evidence on analgesics used to manage dental implant surgery postoperative pain, to identify best therapeutic protocols and knowledge gap. A comprehensive search was conducted including MEDLINE/Pubmed, EMBASE, SCOPUS, clinicaltrials.gov, and the Cochrane Database of Systematic Reviews through May 2020. Only randomized controlled trials were included. PRISMA guidelines were followed, and risk of bias was appraised using Cochrane RoB2 tool. Eleven trials (762 patients overall) were included. Some aspects limited the feasibility of a meaningful meta-analysis; thus, a narrative synthesis was conducted. Risk of bias was low in four studies and high in two studies, while five studies raised some concerns due to the randomization process. Analgesic use seemed to be associated with improved postoperative outcomes (pain, patient’s satisfaction, and need for rescue medication) when compared to placebo. Overall, this review suggests that the administration of analgesics may provide some advantages in the management of postoperative outcomes after dental implant placement, while indications about the best analgesics cannot be provided.

## Introduction

Dental implant therapy has been a revolution in dentistry. Today, oral rehabilitation of single or multiple edentulism with dental implants is a very common procedure, and its use has steadily increased in recent decades (Buser et al., 2017). While intraoperative pain can be effectively controlled with anesthetic agents (Haas, 2002; Bahammam et al., 2017), postoperative pain remains a possible side effect of dental surgery (Wang et al., 2019).

After dental implant placement surgery, patients may present different degrees of postoperative discomfort. Pain and swelling are common consequences of the surgical trauma, induced by the release of inflammatory mediators (Bryce et al, 2014). This could be influenced by various intervention-related factors (such as type of surgery, duration, and extension) and patient characteristics (such as stress level, blood pressure, heart rate, and anxiety) (Scott and Hirschman, 1982).

Pain is usually mild or moderate, although some patients may experience severe pain (Wang et al., 2019). Several drugs (including analgesics, anti-inflammatories, and anesthetics) and different protocols for the management of postoperative pain have been investigated so far, but the literature offers a heterogeneous and undefined picture about effectiveness and treatment-associated adverse events (Bryce et al., 2014).

This systematic review aims to summarize available evidence on analgesics in the management of postoperative pain after dental implant placement.

## Methods

### Study Design

This is a systematic review of randomized controlled trials (RCTs) evaluating analgesic drugs in the management of postoperative pain after dental implant placement. The review was performed following the Preferred Reporting Items for Systematic Reviews and Meta-Analyses (PRISMA) guidelines (Moher et al., 2009). The protocol was registered in PROSPERO (Aug 28, 2020 ID: CRD42020193876).

### Search Strategy

To identify relevant studies, we systematically searched MEDLINE/PubMed, EMBASE, SCOPUS, Cochrane Central Register of Controlled Trials, and Clinicaltrials.gov. The search strategy was carried out without language restrictions from database inception until June 2019. Two investigators (MM and AF) independently reviewed the search results and screened the titles and abstracts. We obtained the full texts of all potentially eligible studies. In PubMed, the following search strategy was used: “((((“dental implants”[MeSH Terms] OR (“dental”[All Fields] AND “implants”[All Fields])) OR “dental implants”[All Fields]) OR (“dental”[All Fields] AND “implant”[All Fields])) OR “dental implant”[All Fields]) AND ((((((“analgesic s”[All Fields] OR “analgesically”[All Fields]) OR “analgesics” [Pharmacological Action]) OR “analgesics”[MeSH Terms]) OR “analgesics”[All Fields]) OR “analgesic”[All Fields]) OR (((“analgesia”[MeSH Terms] OR “analgesia”[All Fields]) OR “analgesias”[All Fields]))).” This search strategy was adapted to suit the other electronic sources. We also hand-searched the reference lists of retrieved articles to identify additional studies of interest. Any inconsistencies were resolved by consensus with a third investigator (MP).

### Criteria for Considering Studies for This Review

Study design: parallel and crossover RCTs.

Population: adult patients (aged 16 or more) undergoing single or multiple dental implant surgeries.

Intervention: any analgesic drugs, defined as compounds capable of relieving pain without the loss of consciousness.

Comparator: any analgesic drugs or placebo.

Outcome: intensity of postoperative pain, swelling, patient’s satisfaction, need for rescue medication, and adverse events.

Time: postoperative.

Studies not including human subjects were excluded. No language restrictions were applied.

### Data Collection

Two investigators (FC and MP) independently extracted key data from the included articles. The inter-rater agreement was assessed using Cohen’s kappa statistics. For each article, we extracted study features (i.e., study design, year of publication, country, number, and age of enrolled patients), type of intervention, and outcomes measures. A third investigator (GZ) checked the extracted data.

### Assessment of Risk of Bias

Two investigators (FC and MP) independently appraised the risk of bias of the included studies by using the Cochrane revised tool to assess risk of bias in randomized trials (RoB 2.0) (Sterne et al., 2019). Five specific domains related to risk of bias of RCTs were assessed (bias arising from the randomization process, bias due to deviations from the intended interventions, bias due to missing outcome data, bias in the measurement of the outcome, and bias in the selection of the reported results). For each domain, a study could be judged to be at low risk of bias, at high risk of bias, or to raise some concerns. Overall, a study was judged to be at low risk of bias if it was at low risk of bias for all domains. It was judged to raise some concerns if it raised some concerns in at least one domain, but was not at high risk of bias for any domain. It was judged to be at high risk of bias if it was at high risk of bias in at least one domain, or raised some concerns for multiple domains in a way that substantially lowers confidence in the results (Sterne et al., 2019). Any inconsistencies were resolved by consensus with a third investigator (MM).

### Data Synthesis

A narrative synthesis of included studies was conducted, because some aspects limited the feasibility of a meaningful meta-analysis. Such aspects included the large number of type analgesic drugs that were evaluated, the heterogeneous outcome measures, and the heterogeneous timing of assessment. The findings from each study were summarized in tables using a narrative approach rather than a quantitative approach, to improve clarity and readability for the readers.

## Results

### Search Results

**FIGURE 1:**
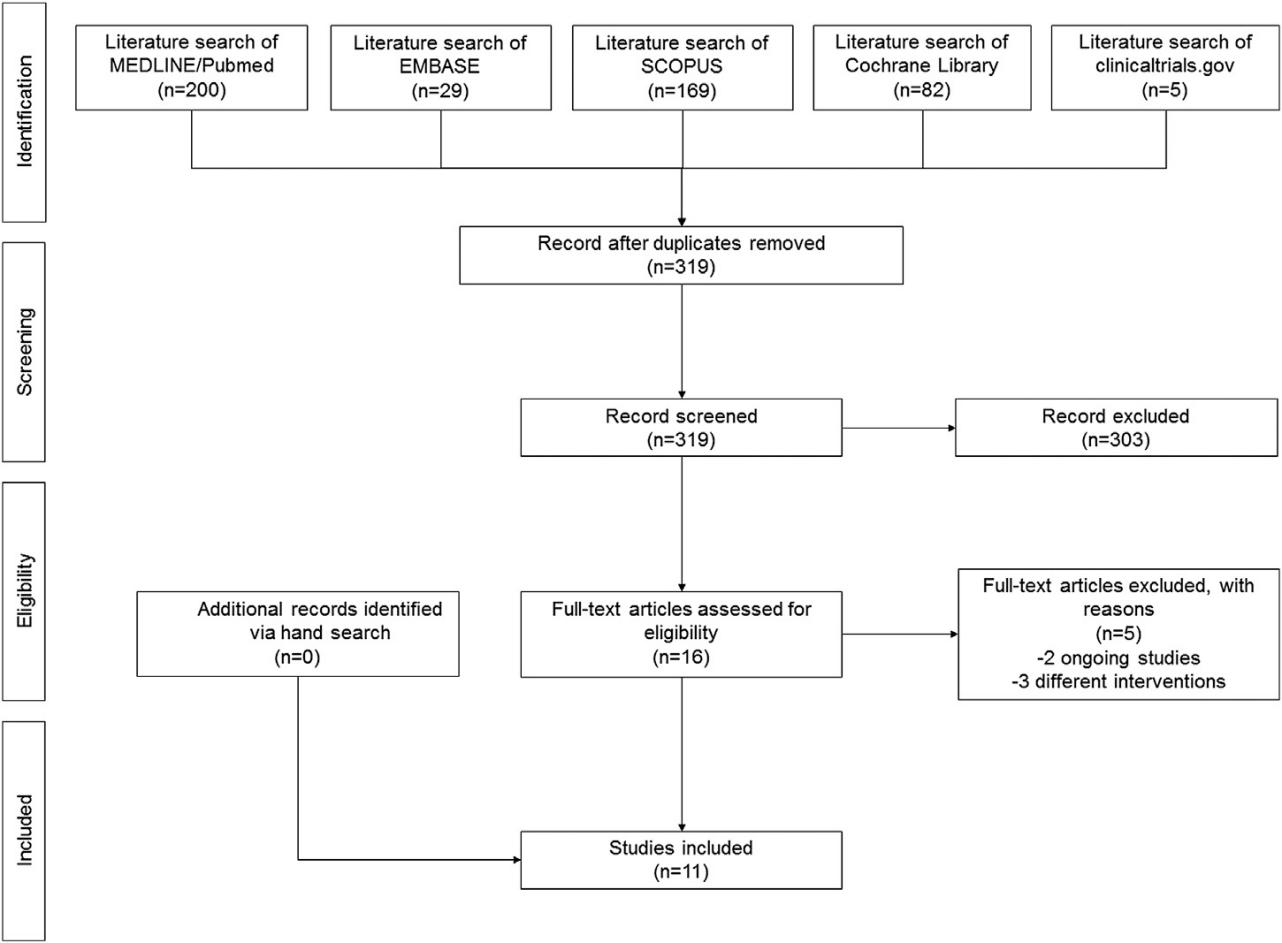
PRISMA flow diagram.

The search yielded 319 non-duplicated articles. After excluding 303 articles based on title/abstract, 16 articles were retrieved for full-text review. Of these, five were excluded due to different interventions (three studies) or unavailability of results (two ongoing studies). No additional articles were identified *via* hand search; thus, a total of 11 RCTs (Pereira et al., 2020; Bhutani et al., 2019; Iero et al., 2018; Sánchez-Pérez et al., 2018; Bahammam et al., 2017; Meta et al., 2017; Samieirad et al., 2017; Raja Rajeswari et al., 2017; Li et al., 2015; Bölükbasi et al., 2012; Karabouda et al., 2007) were included in the qualitative synthesis (Figure 1). Cohen’s kappa indicated a substantial inter-rater agreement (kappa 0.72).

### Study and Patient Characteristics

The analysis included 10 parallel RCTs [Pereira et al., 2020; Bhutani et al., 2019; Iero et al., 2018; Sánchez-Pérez et al., 2018; Bahammam et al., 2017; Meta et al., 2017; Samieirad et al., 2017; Li et al., 2015; Bölükbasi et al., 2012; Karabouda et al., 2007) and one crossover RCT (Raja Rajeswari et al., 2017). Characteristics of included studies are reported in Table 1. The number of enrolled participants ranged from 20 to 117 participants. The type of analgesic drugs included ibuprofen, piroxicam, liposomal bupivacaine, dexketoprofen trometamol, dexamethasone, ketorolac, ketorolac + betamethasone, caffeine, codeine, diclofenac diethylamine, diclofenac sodium, midazolam + fentanyl, dexmedetomidine + fentanyl, lornoxicam, meloxicam, and teloxicam. The timing of administration varied from 24 h before surgery to 72 h after surgery. Outcome measures of interest included postoperative pain (11 studies) (Pereira et al., 2020; Bhutani et al., 2019; Iero et al., 2018; Sánchez-Pérez et al., 2018; Bahammam et al., 2017; Meta et al., 2017; Samieirad et al., 2017; Raja Rajeswari et al., 2017; Li et al., 2015; Bölükbasi et al., 2012; Karabouda et al., 2007), swelling (two studies) (Bhutani et al., 2019; Samieirad et al., 2017), patient’s satisfaction (four studies) (Iero et al., 2018; Bahammam et al., 2017; Raja Rajeswari et al., 2017; Bölükbasi et al., 2012; Bahammam et al., 2017; Raja Rajeswari et al., 2017; Bölükbasi et al., 2012; Karabouda et al., 2007), and need for rescue medication (six studies) (Pereira et al., 2020; Iero et al., 2018; Bahammam et al., 2017; Raja Rajeswari et al., 2017; Bölükbasi et al., 2012; Karabouda et al., 2007). Occurrence of adverse events was reported in six studies (Pereira et al., 2020; Iero et al., 2018; Sánchez-Pérez et al., 2018; Bahammam et al., 2017; Raja Rajeswari et al., 2017; Bölükbasi et al., 2012).

**TABLE 1:** Characteristics of included studies.

#	Study	Country	Study design	Enrolled participants, no	Participant age, years	Implants	Types of analgesic drugs	Timing of administration of analgesic drugs	Outcome measures of interest
1	Pereira et al. (2020)	Brazil	Parallel RCT	54	37–74	Single	Ibuprofen vs. placebo	1 h before surgery	Postop pain (VAS), need for rescue medication, adverse events
2	Bhutani et al. (2019)	India	Parallel RCT	40	16–40	Single	Piroxicam vs. placebo	1 h before surgery	Postop pain (VAS), swelling (using the distance between the lateral corner of the eye and the angle of the mandible and the distance between the tragus of the ear and the outer corner of the mouth)
3	Iero et al. (2018)	United States	Parallel RCT	69	≥18	Full-arch	Standard care + liposomal bupivacaine vs. standard care	At the end of surgery	Postop pain (0–10 scale), patient’s satisfaction (1–5 scale), need for rescue medication, adverse events
4	Sánchez-Pérez et al. (2018)	Spain	Parallel RCT	100	≥18	Single	Dexketoprofen trometamol vs. placebo	15 min before surgery	Postop pain (VAS), adverse events
5	Bahammam et al. (2017)	Kingdom of Saudi Arabia	Parallel RCT	117	≥18	Single	Ibuprofen vs. dexamethasone vs. Placebo	1 h before surgery + 6 h after the first dose	Post pain (VAS, NR101), patient’s satisfaction (VRS-4), need for rescue medication, adverse events
6	Meta et al. (2017)	Argentina	Parallel RCT	30	40–85	Multiple	Ketorolac vs. ketorolac + betamethasone	Within 2 h before surgery	Postop pain (VAS)
7	Samieirad et al. (2017)	Iran	Parallel RCT	80	35–55	Single	Caffeine vs. Codeine	1 h before surgery + every 6 h until 48 h	Postop pain (VAS), swelling (based on VAS)
8	Raja Rajeswari et al. (2017)	India	Crossover RCT	20	30–65	Single	Diclofenac diethylamine transdermal patches vs. Oral diclofenac sodium	After surgery for 72 h	Postop pain (NRS, VRS, PRS), patient’s satisfaction (preferred treatment), adverse events
9	Li et al. (2015)	China	Parallel RCT	60	19–60	Multiple	Midazolam + fentanyl vs. dexmedetomidine + fentanyl	Peri-operative	Postop pain (VAS)
10	Bölükbasi et al. (2012)	Turkey	Parallel RCT	92	18–65	Single/multiple	Lornoxicam vs. Placebo	After surgery	Postop pain (0–3 scale), patient’s satisfaction (1–7 scale), need for rescue medication, adverse events
11	Karabouda et al. (2007)	Turkey	Parallel RCT	100	Mean 53	Multiple	Meloxicam vs. teloxicam	1 day before surgery + 1 h before surgery + for 2 days after surgery	Postop pain (VAS), need for rescue medication

In Iero et al. (2018), standard care included standard care is described as local infiltration at the surgical site with ≤40 ml lidocaine 2% with epinephrine at the beginning of surgery (nerve block); local infiltration at the surgical site with seven carpujects of bupivacaine 0.5% with epinephrine (three mandibular and four maxillary near the end of surgery), ibuprofen (600 mg every 6 h), and oxycodone 5 mg tablets (1 to 2 tablets every 6 h as needed for severe pain).

### Risk of Bias in Included Studies

**FIGURE 2:**
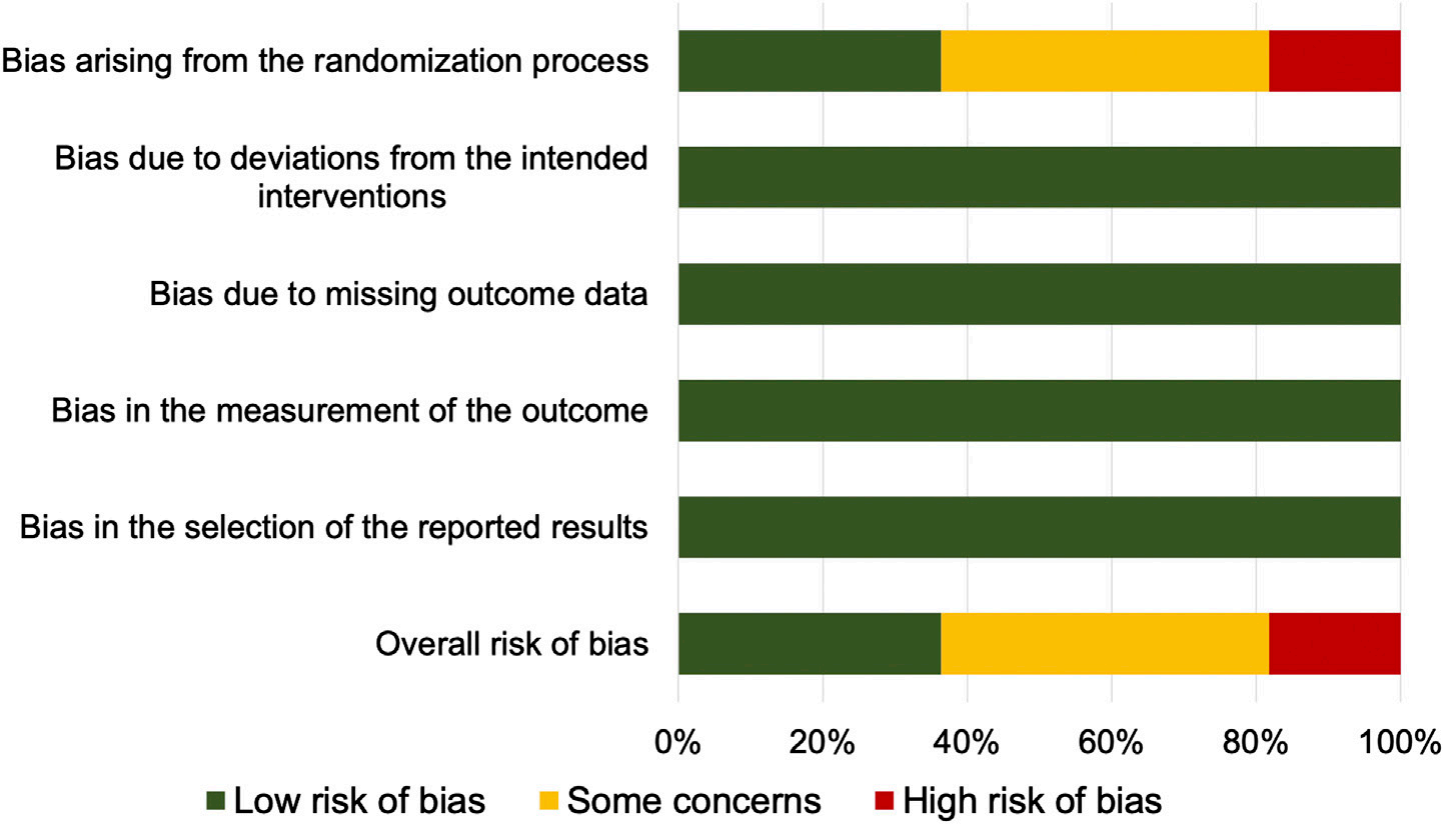
Summary of risk of bias.

The risk of bias is reported in Figure 2. Four studies [Sánchez-Pérez et al., 2018; Samieirad et al., 2017; Bölükbasi et al., 2012; Karabouda et al., 2007) were at low risk of bias for all domains and were judged to be at low overall risk of bias. Five studies (Pereira et al., 2020; Iero et al., 2018; Bahammam et al., 2017; Meta et al., 2017; Li et al., 2015) raised some concerns due to risk of bias arising from the randomization process (unclear information about concealment of allocation sequence and/or presence of baseline imbalances). One study (Bhutani et al., 2019) was at high risk of bias arising from the randomization process (allocation sequence was not concealed) and was judged to be at high overall risk-of-bias. One study (Raja Rajeswari et al., 2017) was at high risk of bias arising from the randomization process (unclear information about concealment of allocation sequence, equality of participants allocated to each sequence, and testing for period effect) and at high risk of bias due to deviations from the intended interventions (participants and personnel were aware of the intervention; no information about deviations from intended intervention and carryover effect), and was judged to be at high overall risk of bias.

### Narrative Synthesis on Postoperative Pain

All eleven studies investigated postoperative pain as reported by patients using visual analogue scale (VAS) [Pereira et al., 2020; Bhutani et al., 2019; Sánchez-Pérez et al., 2018; Bahammam et al., 2017; Meta et al., 2017; Samieirad et al., 2017; Li et al., 2015; Karabouda et al., 2007) or ordered scales (Iero et al., 2018; Raja Rajeswari et al., 2017; Bölükbasi et al., 2012) (Table 2). Lower postoperative pain was reported with ibuprofen vs. placebo (Pereira et al., 2020; Bahammam et al., 2017), piroxicam vs. placebo (Bhutani et al., 2019), standard care + liposomal bupivacaine vs. standard care (Iero et al., 2018), dexketoprofen trometamol vs. placebo (Sánchez-Pérez et al., 2018), dexamethasone vs. placebo (Bahammam et al., 2017), codeine vs. caffeine (Samieirad et al., 2017), dexmedetomidine + fentanyl vs. midazolam + fentanyl (Li et al., 2015), and lornoxicam vs. placebo (Bölükbasi et al., 2012). No statistically significant difference was reported between ibuprofen and dexamethasone (Bahammam et al., 2017), ketorolac vs. ketorolac + betamethasone (Meta et al., 2017), and meloxicam vs. teloxicam (Karabouda et al., 2007). Inconclusive results were reported in one split-mouth study (Raja Rajeswari et al., 2017) because unpaired data analysis was applied to paired data (and published information was not sufficient to redo the analysis).

**TABLE 2:** Postoperative pain (narrative synthesis).

#	Study	Postoperative pain
1	Pereira et al. (2020)	Lower with ibuprofen vs. placebo (1–24 h postop)
2	Bhutani et al. (2019)	Lower with piroxicam vs. placebo (6 h–5 days postop)
3	Iero et al. (2018)	Lower with standard care + liposomal bupivacaine vs. standard care (0–7 days postop)
4	Sánchez-Pérez et al. (2018)	Lower with dexketoprofen trometamol vs. placebo (immediate postop)
5	Bahammam et al. (2017)	Lower with ibuprofen or dexamethasone vs. placebo, but no statistically significant difference between ibuprofen and dexamethasone (1–3 days postop)
6	Meta et al. (2017)	No statistically significant difference between ketorolac vs. ketorolac + betamethasone (3–14 days postop)
7	Samieirad et al. (2017)	Lower with codeine vs. caffeine (3–6–12 h postop)
8	Raja Rajeswari et al. (2017)	Inconclusive results
9	Li et al. (2015)	Lower with dexmedetomidine + fentanyl vs. midazolam + fentanyl (2–4 h postop)
10	Bölükbasi et al. (2012)	Lower with lornoxicam vs. placebo (0.5–4 h postop)
11	Karabouda et al. (2007)	No statistically significant difference between meloxicam vs. teloxicam (1–7 days postop)

### Narrative Synthesis on Swelling

Two studies (Samieirad et al., 2017; Bhutani et al., 2019) investigated swelling (Table 3). Lower swelling was reported with piroxicam vs. placebo (Bhutani et al., 2019), and caffeine vs. codeine (Samieirad et al., 2017).

**TABLE 3:** Swelling (narrative synthesis).

#	Study	Swelling
2	Bhutani et al. (2019)	Lower with piroxicam vs. placebo (1–5 days postop)
7	Samieirad et al. (2017)	Lower with caffeine vs. codeine (1–3 days postop)

### Narrative Synthesis on Patient’s Satisfaction

Four studies [Iero et al., 2018; Bahammam et al., 2017; Raja Rajeswari et al., 2017; Bölükbasi et al., 2012) investigated the patient’s satisfaction (Table 4). Higher patient’s satisfaction was reported with standard care + liposomal bupivacaine vs. standard care (Iero et al., 2018), ibuprofen vs. placebo (Bahammam et al., 2017), dexamethasone vs. placebo (Bahammam et al., 2017), and lornoxicam vs. placebo (Bölükbasi et al., 2012) in the early postoperative period (12–48 h). No statistically significant difference was reported between ibuprofen and dexamethasone (Bahammam et al., 2017). In a split-mouth study (Raja Rajeswari et al., 2017), the majority of patients preferred transdermal diclofenac diethylamine over oral diclofenac sodium.

**TABLE 4:** Patient’s satisfaction (narrative synthesis).

#	Study	Patient’s satisfaction
3	Iero et al. (2018)	Higher with standard care + liposomal bupivacaine vs. standard care (days 0–1)
5	Bahammam et al. (2017)	Higher with ibuprofen or dexamethasone vs. placebo, but no statistically significant difference between ibuprofen and dexamethasone (1–2 days postop)
8	Raja Rajeswari et al. (2017)	18/20 patients preferred transdermal diclofenac diethylamine over oral diclofenac sodium
10	Bölükbasi et al. (2012)	Higher with lornoxicam vs. placebo (12 h postop)

### Narrative Synthesis on Need for Rescue Medication

Six studies [Pereira et al., 2020; Iero et al., 2018; Bahammam et al., 2017; Raja Rajeswari et al., 2017; Bölükbasi et al., 2012; Karabouda et al., 2007) investigated the need for rescue medication (Table 5). Lower need for rescue medication was reported with ibuprofen vs. placebo (Pereira et al., 2020; Bahammam et al., 2017), dexamethasone vs. placebo (Bahammam et al., 2017), and lornoxicam vs. placebo (Bölükbasi et al., 2012). No statistically significant difference was reported between standard care + liposomal bupivacaine vs. standard care (Iero et al., 2018), ibuprofen and dexamethasone (Bahammam et al., 2017), and meloxicam vs. teloxicam (Karabouda et al., 2007). In a split-mouth study (Raja Rajeswari et al., 2017), the patients did not need rescue medication after transdermal diclofenac diethylamine, but the information was unclear after oral diclofenac sodium.

**TABLE 5:** Need for rescue medication (narrative synthesis).

#	Study	Need for rescue medication
1	Pereira et al. (2020)	Lower with ibuprofen vs. placebo
3	Iero et al. (2018)	No statistically significant difference between standard care + liposomal bupivacaine vs. standard care
5	Bahammam et al. (2017)	Lower with ibuprofen or dexamethasone vs. placebo, but no statistically significant different between ibuprofen and dexamethasone
8	Raja Rajeswari et al. (2017)	0/20 transdermal diclofenac diethylamine vs. unclear in oral diclofenac sodium
10	Bölükbasi et al. (2012)	Lower with lornoxicam vs. placebo
11	Karabouda et al. (2007)	No statistically significant difference between meloxicam vs. teloxicam

### Narrative Synthesis on Occurrence of Adverse Events

Six studies [Pereira et al., 2020; Iero et al., 2018; Sánchez-Pérez et al., 2018; Bahammam et al., 2017; Raja Rajeswari et al., 2017; Bölükbasi et al., 2012) reported the occurrence of adverse events (Table 6). Bleeding was more frequent with dexketoprofen trometamol vs. placebo (Sánchez-Pérez et al., 2018). No statistically significant difference was reported between standard care + liposomal bupivacaine vs. standard care (Iero et al., 2018), and transdermal diclofenac diethylamine vs. oral diclofenac sodium (Raja Rajeswari et al., 2017). No adverse events occurred in three studies (Pereira et al., 2020; Bahammam et al., 2017; Bölükbasi et al., 2012).

**TABLE 6:** Adverse events (narrative synthesis).

#	Study	Adverse events
1	Pereira et al. (2020)	None
3	Iero et al. (2018)	No statistically significant difference between standard care + liposomal bupivacaine vs. standard care
4	Sánchez-Pérez et al. (2018)	More bleeding with dexketoprofen trometamol vs. placebo
5	Bahammam et al. (2017)	None
8	Raja Rajeswari et al. (2017)	No statistically significant difference between transdermal diclofenac diethylamine vs. oral diclofenac sodium
10	Bölükbasi et al. (2012)	None

## Discussion

Overall, this review suggested that analgesic use in dental implant placement could be associated with improved postoperative outcomes (including pain, patient’s satisfaction, and need for rescue medication), whereas indications about the best analgesics could not be provided.

To our knowledge, this is the first systematic review about analgesics in dental implant surgery.

Postoperative pain and patient’s discomfort are common consequences of such surgical procedures, and their postoperative management has gathered the attention of several researchers (White and Kehlet, 2010). The literature includes the investigation of different classes of drug, such as NSAID (ibuprofen, piroxicam, meloxicam, lornoxicam, teloxicam, ketorolac, dexketoprofen trometamol, diclofenac diethylamine), corticosteroids (dexamethasone and betamethasone), opioids (codeine and fentanyl), local anesthetics (liposomal bupivacaine), and Alpha2 adrenergic receptor agonists (dexmedetomidine), benzodiazepine (midazolam) (Pereira et al., 2020; Bhutani et al., 2019; Iero et al., 2018; Sánchez-Pérez et al., 2018; Bahammam et al., 2017; Meta et al., 2017; Samieirad et al., 2017; Raja Rajeswari et al., 2017; Li et al., 2015; Bölükbasi et al., 2012; Karabouda et al., 2007). Unfortunately, such heterogeneity—alongside the different timing of assessment—provides an inconclusive picture about effectiveness and safety of these options.

Overall, several analgesics (ibuprofen, piroxicam, dexketoprofen trometamol, dexamethasone, and lornoxicam) were superior in reducing postoperative pain when compared to placebo. (Pereira et al., 2020; Bhutani et al., 2019; Sánchez-Pérez et al., 2018; Bahammam et al., 2017; Bölükbasi et al., 2012). In addition, liposomal bupivacaine showed better analgesic effect when associated with an opioid-sparing postoperative pain management protocol [#3]. These findings are in broad agreement with the literature on postoperative pain relief after third molar extraction surgery, a wider used pain model in dentistry (Filho et al., 2020), o (Weil et al., 2007; Bailey et al., 2013). On the other hand, the comparisons of different analgesics often failed to provide suggestions about the best option for reducing postoperative pain (Bahammam et al., 2017; Meta et al., 2017; Karabouda et al., 2007). Samiera et al. found codeine superior to caffeine in postoperative pain relief, but caffeine was associated with reduced swelling (Samieirad et al., 2017). Li et al. reported lower postoperative pain with dexmedetomidine than midazolam, both in association with fentanyl (Li et al., 2015).

However, NSAIDs have a well-known effect in reducing pain (Bryce et al., 2014). The literature offers controversial findings about its influence on bone regeneration around implants. Gomes et al. found no impairment in osseointegration with COX-1 inhibitors (both in short- and long-term administration), but their safe use during the postoperative period has not been demonstrated (Gomes et al., 2015).

Corticosteroids are usually administered to reduce the inflammatory response after oral surgery (Bryce et al., 2014). While their effectiveness in reducing swelling and trismus after third molar extraction is well accepted, controversial results remain on their direct analgesic properties (Filho et al., 2008; Dionne et al., 2003). Further research on different molecules at different dosages needs to be performed to shed light on this aspect. In our review, dexamethasone 4 mg 1 h before surgery plus 4 mg 6 h after resulted in higher pain reduction than placebo, with no significant difference compared to ibuprofen 600 mg administered at the same time (Bahammam et al., 2017). On the other hand, betamethasone 3 mg EV administered in association with ketorolac did not influence the relief of post-implant pain (Meta et al., 2017). Glucocorticosteroids administered for systemic diseases seem to have no impact on the osseointegration and survival of dental implants placed without bone grafting (Petsinis et al., 2017).

Opioids (such as codeine and fentanyl) have well-known analgesic effects, but other aspects (side effects, abuse, and dependency) should be considered when administered for postsurgical pain. They should be prescribed only when an alternative therapy is not possible or effective, and only for a short period of time (Moore et al., 2015; Eliav, 2017).

Liposomal bupivacaine is a local anesthetic formulation consisting of bupivacaine hydrochloride encapsulated within multiple nonconcentric lipid bilayers, in order to offer sustained-release analgesia (Hamilton et al., 2017). Hamilton et al. suggested that its infiltration at the surgical site may reduce postoperative pain when compared to placebo but could not demonstrate superiority to bupivacaine hydrochloride (low-quality evidence) (Hamilton et al., 2017).

Dexmedetomidine is an α_2_-adrenoreceptor agonist with sedative, anxiolytic, sympatholytic, and analgesic-sparing effects, and minimal depression of respiratory function. Analgesic effects of α_2_-agonists are thought to be mediated by α_2_-receptor binding on central and spinal cord α_2_-receptors. Pain transmission is suppressed by hyperpolarization of interneurons and reduction of the release of pronociceptive transmitters such as substance P and glutamate (Weerink et al., 2017). The mechanisms underlying the analgesic effects of dexmedetomidine are still incompletely understood and may partly be owing to an altered perception and reduced anxiety, although an opioid-sparing effect is described, and there may be an effect when used with locoregional anesthesia techniques (Weerink et al., 2017).

Midazolam is a benzodiazepine characterized by rapid onset of clinical effects and short duration of action; like other benzodiazepines, its pharmacological action includes sedation, sleep induction, anxiolysis, and amnesia (Nordt and Richard Clark, 1997). Its antinociceptive effect is still unclear; some authors report significant results in animal models (Chiba et al., 2009; Guo and Yuan, 2008; Kyles et al., 1995), while other studies report no influence on pain reduction when associated with other sedatives or analgesics (Auffret et al., 2014; Li et al., 2015; Cai et al., 2017).

The use of caffeine (>100 mg) as an adjunct to common analgesics has been reported to provide a small but important increase (5–10%) in the proportion of patients who experience pain relief (Derry et al., 2014). Our review found it effective in the reduction of swelling when compared to codeine, a result also observed with piroxicam when compared to placebo (Buthani et al., 2019; Samieirad et al., 2017).

Patients usually report high satisfaction about dental implants, with some influence of pre-operatory anxiety and prosthetic complications over time (Al-Radha, 2019; Canallatos et al., 2020). Improved patient’s satisfaction was associated with some analgesics (liposomal bupivacaine, ibuprofen, dexamethasone, and lornoxicam) compared to placebo (Iero et al., 2018; Bahammam et al., 2017; Bölükbasi et al., 2012), while ibuprofen and dexamethasone achieved comparable results (Bahammam et al., 2017). In one split-mouth study (Raja Rajeswari et al., 2017), participants preferred transdermal diclofenac diethylamine over oral diclofenac sodium, but the findings could have been biased due to the randomization process and the deviations from the intended interventions. Within the limitations of this review, the administration of analgesics appears to improve overall patient’s satisfaction about dental implant surgery.

Rescue medication includes drugs that may be administered to the patient when the efficacy of the investigational medical product is not satisfactory (Nahle, 2009). Reduced need for rescue medication was achieved with ibuprofen (Pereira et al., 2020; Bahammam et al., 2017), dexamethasone (Bahammam et al., 2017), and lornoxicam (Bölükbasi et al., 2012) compared to placebo. Analgesics may have a wide variety of adverse effects, from mild to severe, that should be taken into account when analgesic therapy is prescribed (Kim and Seo, 2020).

No adverse events occurred when using ibuprofen, dexamethasone, and lornoxicam (Pereira et al., 2020; Bahammam et al., 2017; Bölükbasi et al., 2012); side effects reported with liposomal bupivacaine were comparable to the standard of care (Iero et al., 2018); and a higher incidence of bleeding was reported in patients receiving dexketoprofen trometamol than in those receiving placebo (Sánchez-Pérez et al., 2018). When analgesics are prescribed, evaluating a patient’s medical history, severity of patient’s expected pain, pharmacological properties of the drugs, and potential interactions with concurrent medications become crucial in order to reduce the occurrence of adverse events.

The findings of this review should be interpreted within its limitations. First, the heterogeneity in analgesic drugs and timing of assessment precluded the pooling of the results, thus limiting the summary of the findings to a narrative synthesis. Second, the mixed quality of the included studies and the small number of studies investigating each outcome prevented from drawing strong conclusions.

Nonetheless, this review suggests that the administration of analgesics may provide some advantages in the management of postoperative pain after dental implant placement, but further research is warranted. While the available literature offers some analgesic protocols for dental pain based on anticipated postprocedural pain level (ADA 2020; Kim and Seo, 2020), specific evidence-based analgesic schemes for dental implant surgery remain undefined. Unfortunately, the wide surgical variability of the implantology practice and the large quantity of molecules and protocols available in the literature prevent from providing indications about the best treatment for postoperative pain control. Further research including studies with adequate sample size comparing standardized implant approaches is needed to inform best practices in this domain.

## Data Availability Statement

The original contributions presented in the study are included in the article/Supplementary Material, and further inquiries can be directed to the corresponding author.

## Author Contributions

Conception or design of the work: all authors. Acquisition of data: MM, AF, and GZ. Analysis and interpretation of data: FC and MP. Draft of the work: MM, AF, and FC. Revision of the manuscript for important intellectual content: MP and GZ. All authors approved the submitted version and agreed to be accountable for the submitted work.

## Conflict of Interest

The authors declare that the research was conducted in the absence of any commercial or financial relationships that could be construed as a potential conflict of interest.
